# Enhanced production of clavulanic acid by improving glycerol utilization using reporter-guided mutagenesis of an industrial *Streptomyces clavuligerus* strain

**DOI:** 10.1093/jimb/kuab004

**Published:** 2021-01-25

**Authors:** Chang-Hun Shin, Hang Su Cho, Hyung-Jin Won, Ho Jeong Kwon, Chan-Wha Kim, Yeo Joon Yoon

**Affiliations:** Department of Biotechnology, College of Life Sciences and Biotechnology, Korea University, Seoul 02841, Republic of Korea; Department of Biotechnology, College of Life Science and Biotechnology, Yonsei University, Seoul 03722, Republic of Korea; Fermentation Technology Team, Research Institute of CKD Bio, Ansan 15604, Republic of Korea; Department of Biotechnology, College of Life Science and Biotechnology, Yonsei University, Seoul 03722, Republic of Korea; Department of Biotechnology, College of Life Sciences and Biotechnology, Korea University, Seoul 02841, Republic of Korea; Natural Products Research Institute, College of Pharmacy, Seoul National University, Seoul 08826, Republic of Korea

**Keywords:** Clavulanic acid, *Streptomyces clavuligerus*, Glycerol utilization, Reporter-guided selection

## Abstract

Clavulanic acid (CA) produced by *Streptomyces clavuligerus* is a clinically important β-lactamase inhibitor. It is known that glycerol utilization can significantly improve cell growth and CA production of *S. clavuligerus*. We found that the industrial CA-producing *S. clavuligerus* strain OR generated by random mutagenesis consumes less glycerol than the wild-type strain; we then developed a mutant strain in which the glycerol utilization operon is overexpressed, as compared to the parent OR strain, through iterative random mutagenesis and reporter-guided selection. The CA production of the resulting *S. clavuligerus* ORUN strain was increased by approximately 31.3% (5.21 ± 0.26 g/l) in a flask culture and 17.4% (6.11 ± 0.36 g/l) in a fermenter culture, as compared to that of the starting OR strain. These results confirmed the important role of glycerol utilization in CA production and demonstrated that reporter-guided mutant selection is an efficient method for further improvement of randomly mutagenized industrial strains.

## Introduction

β-Lactams, such as penicillin and cephalosporin, are useful antibiotic therapies to combat bacterial infection (Hamed et al., 2013). These antibiotics block enzymes called penicillin-binding proteins that are essential for bacterial cell-wall synthesis. However, the widespread use of β-lactam antibiotics has reduced their efficacy due to the emergence of resistance among invading pathogens. One of the clinically effective ways to overcome resistance to β-lactam antibiotics is the combined use of a β-lactam antibiotic and a β-lactamase inhibitor such as clavulanic acid (CA) (Tahlan & Jensen, 2013), although CA itself exhibits weak antibacterial activity against most bacteria. Commercial products such as Augmentin™ and Timentin™, which are mixtures of CA and conventional β-lactam antibiotics, have been prescribed in many countries (Elander, 2003).

**Fig. 1.**
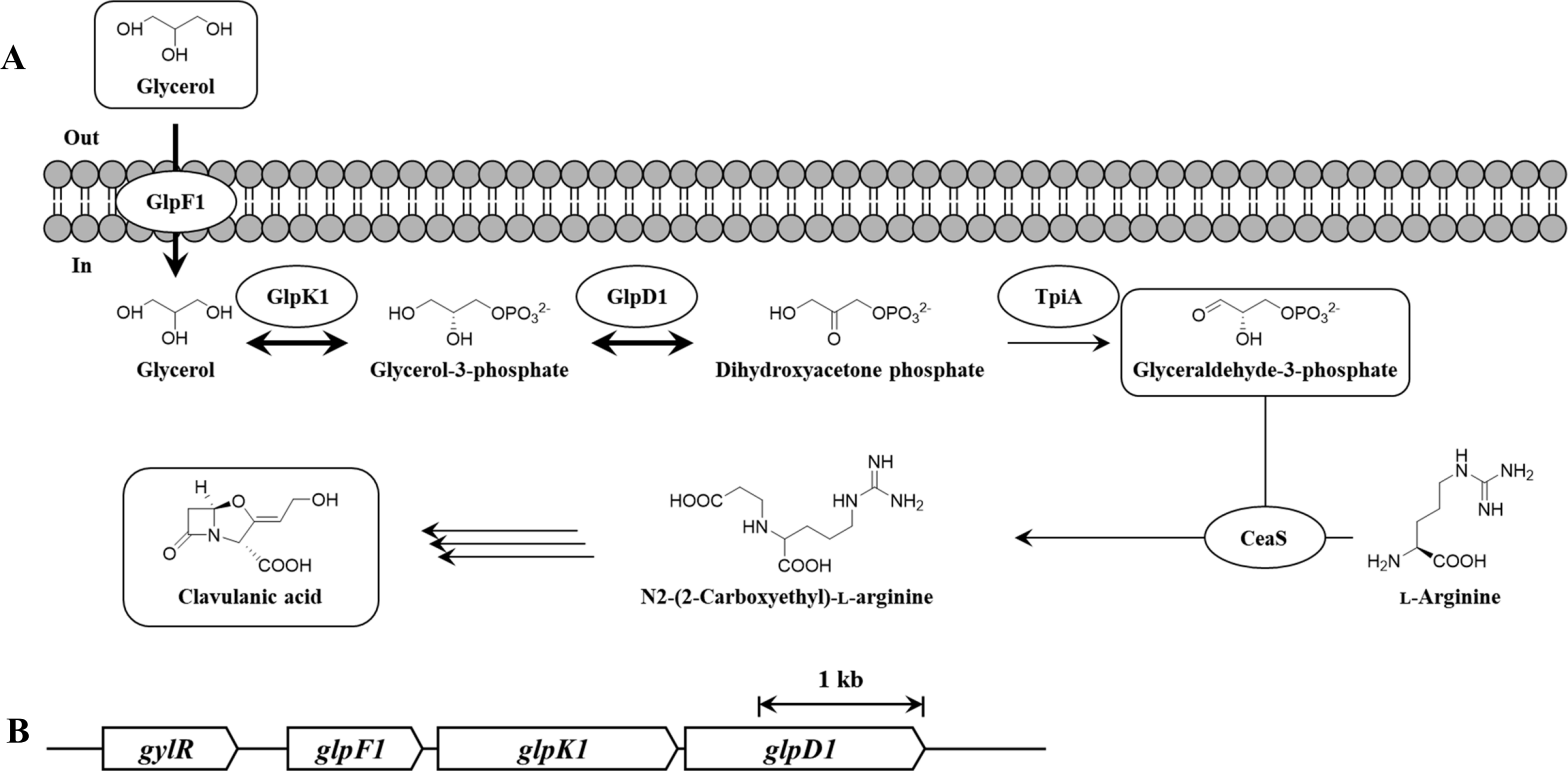
Schematic representation of glycerol utilization and CA biosynthesis in *S. clavuligerus*. (A) Overexpressed glycerol utilization pathway in this study is indicated by bold arrows. GlpF1, glycerol transporter; GlpK1, glycerol kinase; GlpD1, glycerol-3-phosphate dehydrogenase; TpiA, triose phosphate isomerase; CeaS, carboxyethylarginine synthase. (B) Organization of the *glp* operon. *gylR*, IclR family transcriptional regulator.

CA biosynthesis begins with the condensation of l-arginine and glyceraldehyde-3-phosphate (G3P) by carboxyethylarginine synthase (CeaS), which is the first enzyme involved in CA biosynthesis in *Streptomyces clavuligerus* (Hamed et al., 2013; Jensen, 2012) (Fig. 1A). G3P, a direct precursor of CA, is derived from either glucose or glycerol. The importance of glycerol supply in CA biosynthesis has been demonstrated in *S. clavuligerus* (Paradkar, 2013). For instance, glycerol feeding of cultures enhanced CA production by approximately 2-fold (Chen et al., 2003). Moreover, the expression of additional copies of the glycerol utilization operon increased the CA yield by ∼4.5-fold, and further glycerol supplementation enhanced CA production by ∼7.5-fold (Baños et al., 2009). In *S. clavuligerus*, the genes responsible for glycerol utilization are organized in a *glp* operon consisting of *gylR-glpF1-glpK1-glpD1*; the *gylR* gene encodes an IclR family transcriptional regulator, and the *glpF1, glpK1,* and *glpD1* genes code for a putative glycerol transporter, glycerol kinase, and glycerol-3-phosphate dehydrogenase, respectively (Guo et al., 2013). It is known that *gylR* is transcribed as a single transcript, and *glpF1-glpK1-glpD1* is polycistronically expressed under the control of a putative promoter located upstream of *glpF1* (Baños et al., 2009) (Fig. 1B). The *glp* operon is also essential for cell growth on glycerol (Baños et al., 2009). For example, glycerol supplementation improved the growth of a wild-type *S. clavuligerus* strain by approximately 2.5-fold, but the deletion of *glpK1* caused delayed cell growth that corresponded to the growth of the wild-type strain in the absence of glycerol (Baños et al., 2009). In addition, transcriptome analysis of an industrial CA-producing strain obtained through iterative mutagenesis showed that the genes involved in glycerol uptake and metabolism are upregulated in the industrial strain (Medema et al., 2011). Taken together, improved utilization of glycerol through overexpression of the *glp* operon in *S. clavuligerus* can have significant effects on the enhancement of cell growth and CA production.

Many attempts to increase the production of high-value secondary metabolites for strain improvement in an industrial environment have been dependent upon random mutagenesis and selection (Rowlands, 1984). Although this conventional strain development method has been successful for decades (Medema et al., 2011; Ünsaldı et al., 2017), it is a time-consuming and laborious approach for the identification of improved mutants that exist among an enormous mutagenized population. To facilitate the mutant selection process, a semirational method based on the coexpression of a reporter gene and a key biosynthetic gene have been developed. For example, a kanamycin resistance gene (*neo*) fused with the pathway-specific regulator *claR* of CA biosynthesis in *S. clavuligerus* was used as an indicator for high-level expression of *claR*, resulting in the selection of a mutant that produces approximately 33% more CA than the starting strain (Qin et al., 2017). In addition, CA production by a mutant of *S. clavuligerus* generated from the double reporter strategy, in which an *xylE* (a catechol 2,3-dioxygenase gene)-*neo* double reporter system was used to monitor the expression of *ccaR* (another pathway-specific regulator for CA biosynthesis), was increased by approximately 4-fold (Xiang et al., 2009). However, the application of such a reporter strategy to a randomly mutagenized industrial strain of *S. clavuligerus* is limited by the difficulty in the identification of the appropriate target genes among the vast accumulation of unpredictable mutations (Medema et al., 2011; Ünsaldı et al., 2017).

In this work, we first identified that the high-CA-producing *S. clavuligerus* strain OR consumes less glycerol in culture than the wild-type strain, and we subsequently increased glycerol utilization through iterative random mutagenesis and selection using the *neo* gene as a reporter for the increased expression of the *glp* operon. The resulting mutant strain *S. clavuligerus* ORUN showed significantly enhanced expression of the *glp* operon, compared to that of the starting strain (approximately 5.3-fold). CA production by *S. clavuligerus* ORUN was increased by approximately 31.3% (5.21 ± 0.26 g/l) in flask culture and 17.4% (6.11 ± 0.36 g/l) in fermenter culture, as compared to that of the starting OR strain. These results confirmed the significant effect of glycerol utilization on CA production and demonstrated that reporter-guided mutant selection can be a practical strategy for further improvement of randomly mutagenized industrial strains.

## Materials and Methods

### Bacterial Strains and Plasmids

All bacterial strains and plasmids used in this study are listed in Table 1. The high-CA-producing *S. clavuligerus* strain OR (Cho et al., 2019) was generated from *S. clavuligeru*s NRRL 3585 (Kim et al., 2009) through random mutagenesis using ultraviolet (UV) irradiation. *E. coli* HST08 (Takara, Shiga, Japan) was used as the host for general cloning, and non-methylating *E. coli* ET12567/pUZ8002 (Kieser et al., 2000) was used for conjugal transfer of the recombinant plasmid between *Streptomyces* and *E. coli. S. clavuligerus* NRRL 3585 and its derivative mutants were maintained on ISP4 (Difco, Sparks, MD, USA). The temperature-sensitive *E. coli*-*Streptomyces* shuttle vector pKC1139 (Bierman et al., 1992) containing an apramycin resistance marker was used for gene insertion. Authentic CA was provided by CKD Bio Corporation (Ansan, Korea).

**Table 1.** Strains and Plasmids used in this Study

Strains/plasmids	Description^a^	References
***E. coli* strains**		
HST08	Host for general cloning	Takara
ET12567/pUZ8002	Donor strain for intergeneric conjugation between *E. coli* and *Streptomyces*	Kieser et al. (2000)
***Streptomyces* strains**		
*Streptomyces clavuligerus* NRRL 3585	Wild-type clavulanic acid-producing strain	Kim et al. (2009)
*Streptomyces clavuligerus* OR	Industrial high-producing strain	Cho et al. (2019)
*Streptomyces clavuligerus* ORN	*Streptomyces clavuligerus* OR containing *neo* downstream of *glpD*1	This study
*Streptomyces clavuligerus* ORUN	Mutant obtained from *Streptomyces clavuligerus* ORN with increased co-transcription of *glp* operon with *neo*	This study
**Plasmids**		
pKC1139	Temperature-sensitive *E. coli-Streptomyces* shuttle vector containing *oriT* and *aac(3)IV* for gene insertion	Bierman et al. (1992)
pKD04	pKC1139-based plasmid containing *neo* and flanking region	This study

^a^ *neo*: kanamycin resistance gene; *oriT*: origin of transfer; *aac(3)IV*: apramycin resistance gene.

### Construction of Neo Insertion Mutant Strain

**Fig. 2.**
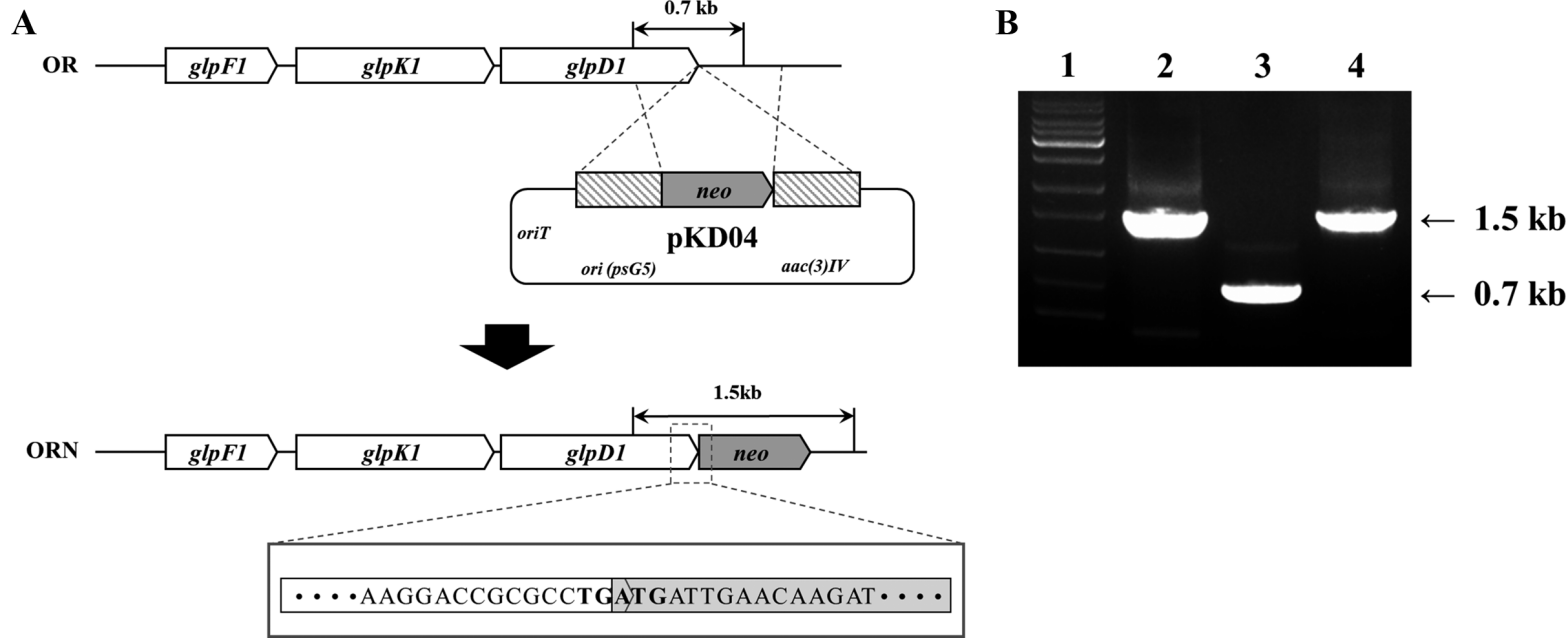
Construction of *neo*-labeled *S. clavuligerus* ORN strain from *S. clavuligerus* OR via homologous recombination. (A) Schematic representation of homologous recombination between *glp* operon and *neo* gene insertion plasmid pKD04. The promoterless kanamycin resistance gene *neo* is fused downstream of *glpD1*. Partial sequences of *glpD1* and *neo* are shown to indicate the overlapping TGA stop codon and ATG start codon. (B) PCR verification of ORN strain. **1**, Marker; **2**, pKD04; **3**, OR strain; **4**, ORN strain.

To construct the plasmid for the insertion of the *neo* gene downstream of the *glp* operon, the promoterless *neo* gene (GenBank accession no. V00618.1; 151 nt–945 nt) including the left flanking region (GenBank accession no. CP027858.1; 5 645 566 nt–5 646 065 nt) and right flanking region (GenBank accession no. CP027858.1; 5 645 066 nt–5 645 565 nt) was synthesized by Macrogen Corporation (Seoul, Korea) (Fig. S1). The synthesized DNA fragment was digested with *Hind*III and *Xba*I and cloned into pKC1139, resulting in pKD04. The *neo* insertion plasmid pKD04 was introduced into *S. clavuligerus* OR by conjugation from *E. coli* ET12567/pUZ8002 (Kieser et al., 2000), and *neo* was then inserted into the genome by homologous recombination. The *neo* gene was fused to *glpD1* by overlapping its start codon with the stop codon of *glpD1* (Fig. 2A). The desired double-crossover mutant ORN was selected by its apramycin sensitive phenotype as described previously (Mo et al., 2011) and verified by PCR using primers GK-check-forward (5′-CCGCTGCCCGGTATAGCCAA-3′) and GK-check-reverse (5′-CGCACATCCAGAATCCGGAT-3′).

### Mutagenesis and Reporter-Guided Selection

A combination of UV irradiation and *N*-methyl-*N*′-nitro-*N*-nitrosoguanidine (MNNG) was used in the two-step mutagenesis for the selection of a mutant with increased cotranscription of the *glp* operon with *neo* from the ORN strain. The intensity of UV light causing 99.9% lethality and the dosage of MNNG treatment causing 90% lethality were chosen for the mutagenesis. First, mature spores of ORN were mutagenized by UV irradiation. The mutagenized spore suspensions were then diluted and spread onto minimal medium plates containing 0.1% glycerol, 0.1% CaCO_3_, 0.01% (NH_4_)_2_SO_4_, 0.06% K_2_HPO_4_, 0.01% MgSO_4_·7H_2_O, 0.03% NaCl, 0.0003% FeCl_3_·6H_2_O, 0.0005% MnSO_4_·H_2_O, 0.0005% CuCl_2_·2H_2_O, 0.0005% ZnCl_2_, 1% agar, and 1.5 μg/ml kanamycin. Kanamycin-resistant mutant colonies were randomly selected and directly inoculated into sterile 96-well plates with a high-throughput screening (HTS) medium containing 4% glycerol, 1.5% soy peptone, 1.05% 4-morpholinepropanesulfonic acid (MOPS), 0.12% KH_2_PO_4_, 0.01% CaCl_2_·2H_2_O, 0.01% MgSO_4_·7H_2_O, 0.003% FeCl_3_·6H_2_O, 0.0005% MnSO_4_·H_2_O, 0.0005% CuCl_2_·2H_2_O, and 0.0005% ZnCl_2_ with the automated colony picking device Qpix2 (Genetix, San Jose, CA, USA) (Song et al., 2010). All mutant strains were cultivated at 28°C with shaking at 1200 rpm for 6 days prior to CA titer testing. The relative CA production was measured using a modified procedure of a simple spectrophotometric assay, as described previously (Bird et al., 1982). The culture broth in each well was diluted by adding 500 μl of acetate buffer (pH 6.6). After centrifugation of the 96-well plates at 3000 rpm for 15 min, 10 μl of the supernatant from each well was transferred to a new 96-well plate and diluted by the addition of 40 μl of acetate buffer. After adding 2% imidazole solution (pH 6.8), the relative CA titers of 1056 mutants were determined at 313 nm (Bird et al., 1982). The top 20 mutant strains showing high CA yield were selected and additionally mutagenized by MNNG (Rowlands, 1984). The mutagenized spores were plated onto a minimal medium agar containing 2.0 μg/ml of kanamycin, and the ability of the 1,056 randomly selected mutants to produce CA was evaluated on HTS plates by the spectrophotometric assay as described above. The mutant strain that displayed the highest CA titer was selected and designated as strain *S. clavuligerus* ORUN.

### RNA Extraction and Gene Expression Analysis by qRT-PCR

In order to confirm the gene expression change of the *glp* operon by reporter-guided mutation, total RNA was isolated from *S. clavuligerus* strains ORN and ORUN grown in production medium (3% glycerol, 4.7% soy flour, 1.05% MOPS, 0.16% NaH_2_PO_4_, 0.01% NaCl, 0.01% MgSO_4_·7H_2_O, 0.004% FeCl_3_·6H_2_O, 0.0005% MnSO_4_·H_2_O, 0.0005% CuCl_2_·2H_2_O, 0.0005% ZnCl_2_) for 16 and 72 h, respectively, as described elsewhere (Jung et al., 2011). The harvested cells were suspended in 1 ml of TRIzol reagent (Invitrogen, Carlsbad, CA, USA) and incubated at room temperature for 5 min. The mixture was vortexed with 200 μl of chloroform and centrifuged at 13 000 × *g* for 15 min. The supernatant was collected in a new tube and mixed with one volume of 70% ethyl alcohol. The mixture was transferred to an RNeasy Mini spin column (Qiagen, Hilden, NRW, Germany) and centrifuged at 8,000 × *g* for 15 s at room temperature. The spin column was washed with RPE washing buffer (Qiagen, Hilden, NRW, Germany), and the resulting total RNA was dissolved in 50 μl of RNase-free water. The nucleic acid preparations were treated with DNase I (New England Biolabs, Ipswich, MA, USA) as recommended by the manufacturer.

The first-strand cDNA was synthesized using a Prime Script 1st strand cDNA Synthesis Kit (Takara, Shiga, Japan). The reaction parameters were as follows: 95°C for 10 min, followed by 35 three-step amplification cycles consisting of denaturation at 95°C for 30 s, annealing at 58°C for 30 s, and extension at 72°C for 30 s. The amplification specificity of each assay was confirmed by melting curve analysis carried out at 60–95°C. After the reaction was run on an Applied Biosystems 7500 Real-Time PCR instrument (Thermo Fisher Scientific, Waltham, MA, USA), the results were analyzed using the supporting 7500 software (v2.0.4). The primers designed for the *glpF1, glpK1, glpD1*, and *neo* are summarized in Table S1, and the primers designed for *hrdB* were used to normalize all transcription values to the internal control as described previously (Li et al., 2015). The experiments were carried out in triplicate using RNA samples from three independent cultures.

### Kanamycin Susceptibility Test

Liofilchem™ MTS™ kanamycin test strips (Liofilchem, Abruzzo, TE, Italy) were used to determine the minimum inhibitory concentrations (MICs) of kanamycin against OR, ORN, and ORUN strains as described previously in order to confirm the increased kanamycin resistance of the mutant strains (Baker et al., 1991). The test was performed according to the manufacturer's instructions. A spore suspension containing 2 × 10^8^ CFU/ml was spread onto a minimal medium agar plate for lawn growth, and the strip with a predefined concentration gradient of kanamycin (256 μg/ml at the top of the strip) was placed on the agar surface. The test plates were cultivated at 28°C for 7 days, and the MICs were determined from the inhibition zone ellipses that intersected the scale indicated on the strips.

### Cultivation for CA Production

A volume of 0.5 ml of spore suspension was inoculated into 20 ml of seed medium containing 3.1% corn starch, 3.2% soy flour, 3% glycerol, 0.5% yeast extract, 0.1% K_2_HPO_4_, 0.05% MgSO_4_·7H_2_O, 0.025% FeSO_4_·7H_2_O, and 0.006% ZnCl_2_ in a 500 ml baffled Erlenmeyer flask that was then cultivated on an orbital shaker at 210 rpm and 25°C for 48 h. A volume of 1 ml of the seed culture was transferred to 50 ml of the production medium described above in a 500 ml baffled Erlenmeyer flask. The main culture was cultivated at 210 rpm and 25°C for 5 days.

For large scale fermentation, the seed culture was incubated at 220 rpm and 28°C for 32 h as described above. The main cultivation was carried out in a 7 l jar fermenter (Korea Fermenter, Incheon, Korea) as described previously (Cho et al., 2019). After a 24 h incubation, the main culture was continuously supplemented with additional glycerol (10 g/l/day).

### Analysis of CA Production

The CA titer in the sample was estimated using high-performance liquid chromatography (HPLC) as reported previously (Cho et al., 2019). The culture broth (0.1 ml) from the main culture was transferred to a 100 ml flask and diluted with 20 mM acetate buffer (pH 6.6), followed by injection with a mixture of 16.6 mM NaH_2_PO_4_ and methanol (86:14, vol/vol) into the HPLC machine equipped with a SUPERCOSIL^TM^ LC-18 HPLC column (5 μm, 150 × 4.6 mm, Sigma-Aldrich, Saint Louis, MO, USA). The flow rate was 1 ml/min, and CA was detected by UV absorbance at 238 nm. Standard solutions of CA at different concentrations were used to generate a calibration curve. The CA production levels were averaged based on five separate cultivations.

### Analysis of Glycerol Consumption

The concentration of glycerol in the sample was analyzed using HPLC. The sample was prepared as described above and injected into the HPLC apparatus according to a modified procedure described previously ( Daskalaki & Kondarides, 2009). HPLC analysis was performed with a SUPERCOGEL^TM^ H, 6% crosslinked HPLC column (9 μm, 250 × 4.6 mm, Sigma-Aldrich, Saint Louis, MO, USA) by the isocratic method using 0.1% H_3_PO_4_ as the solvent. The flow rate was 0.5 ml/min and a refractive index detector was used for glycerol detection. Glycerol standards were used to obtain a calibration curve. The residue levels of glycerol in the cultures were averaged from five separate cultivations.

### Viscosity Measurement

The apparent viscosity of the culture was estimated using a Brookfield viscometer equipped with a small sample adaptor (Model DV-E, Middleboro, MA, USA). Readings were taken using 16 ml of the sample at 30 rpm, 25°C, and spindle number 25. The viscosity was used as a biomass parameter for calculating cell growth (Neves et al., 2000).

## Results and Discussion

### Glycerol Consumption of *S. clavuligerus* OR Mutant

**Fig. 3.**
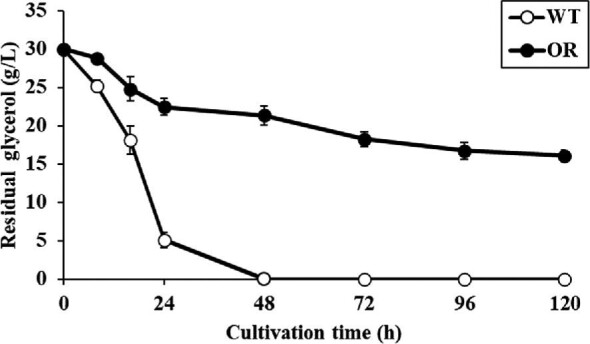
Time course for glycerol consumption in *S. clavuligerus* NRRL 3585 and OR strain. WT, *S. clavuligerus* NRRL 3585.

Our previous study reported that the strain *S. clavuligerus* OR developed through random mutagenesis produces 3.97 ± 0.10 g/l of CA in the flask culture, whereas the wild-type NRRL 3585 strain produces 0.53 ± 0.11 g/l of CA (Cho et al., 2019). The residual amounts of glycerol in the cultures of the wild-type and OR strains were monitored over a 120 h period to investigate whether enhanced glycerol utilization is one of the primary causes for the high CA titer of the OR strain, as has been reported for another randomly mutagenized industrial strain of *S. clavuligerus* (Medema et al., 2011) (Fig. 3). The residual glycerol in the culture of the wild-type strain was notably decreased between 8 and 24 h and exhausted at 48 h. On the other hand, high-CA-producing strain *S. clavuligerus* OR consumed approximately 7.52 g/l of glycerol within 24 h (∼30.2% of the wild-type strain), and the glycerol consumption by this mutant strain was markedly decreased after 24 h; more than half of the glycerol remained in the culture medium after 120 h. This suggested that the improved CA production in the OR strain is not related to the improved glycerol utilization, and the CA production by the OR strain can be further enhanced through overexpression of the *glp* operon responsible for glycerol utilization.

### Generation of ORUN Strain by Reporter-Guided Selection Strategy

The reporter-guided mutant selection approach was designed using *neo* as a reporter gene to improve glycerol utilization in *S. clavuligerus* strain OR. The promoterless *neo* gene was fused to *glpD1* by overlapping its start codon with the stop codon of *glpD1* (Fig. 2A) by double-crossover homologous recombination of the insertion plasmid pKD04. The specific ribosome binding site for *neo* is not required because translational coupling from the upstream gene may reinitiate translation of the downstream *neo* gene, as described previously (Tian & Salis, 2015). The genotype of the resulting *S. clavuligerus* ORN strain was confirmed by colony PCR using the primers GK-check-forward and GK-check-reverse described above (Fig. 2B).

**Fig. 4.**
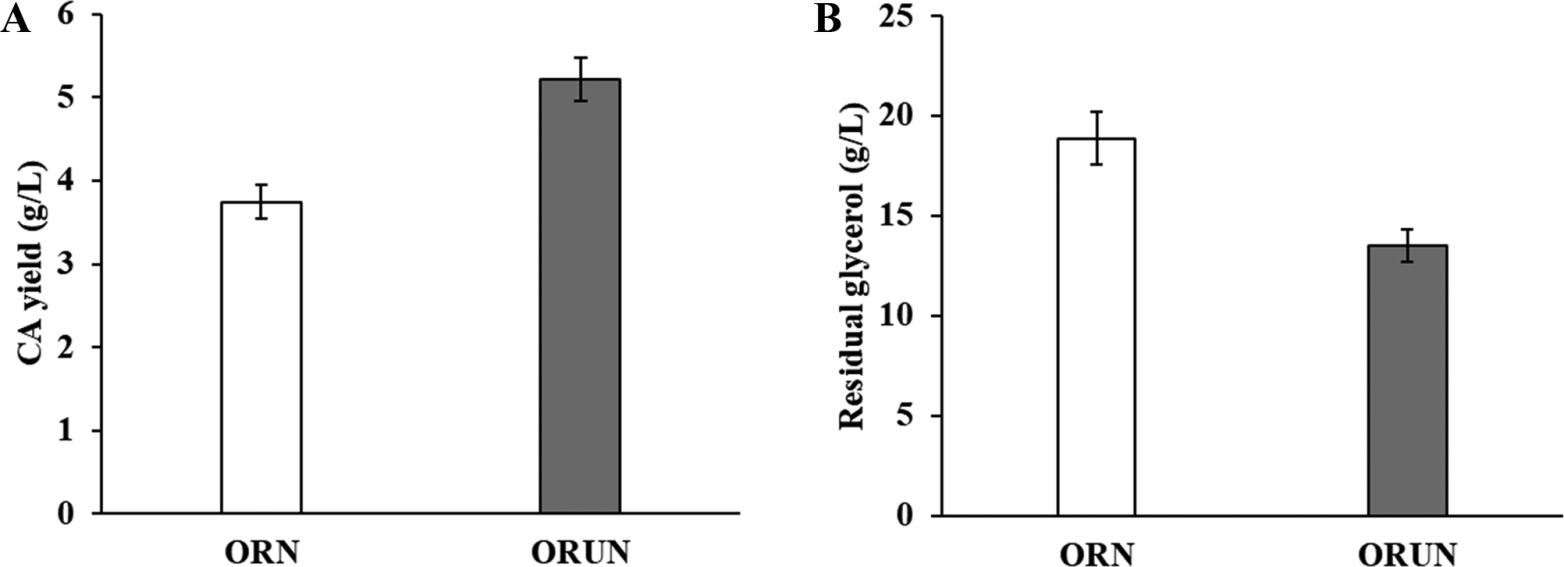
Clavulanic acid production and residual glycerol from *S. clavuligerus* ORN and ORUN after 120 h cultivation in the flask. (A) Clavulanic acid production; (B) residual glycerol.

A two-step mutagenic screening approach was conducted to enhance the expression of the *glp* operon of *S. clavuligerus* ORN. After two-step mutagenic screening, the mutant that produced the maximum CA titer (5.21 ± 0.26 g/l) in a flask culture was selected and designated *S. clavuligerus* ORUN (Fig. 4A). The CA production of the mutant strain ORUN was enhanced by approximately 39.1%, compared to that of the ORN strain. This is comparable to findings of a previous study; the CA titer of *S. clavuligerus* M3–19 was increased by approximately 33% over the starting strain in which the pathway-specific regulator *claR* for CA biosynthesis was overexpressed using *neo* as an indicator (Qin et al., 2017).

In addition, the residual glycerol level in the culture of ORUN (13.5 ± 0.8 g/l) was lower than that in the culture of ORN (18.9 ± 1.31 g/l) after 120 h cultivation. Considering the initial glycerol concentration (30 g/l) in the culture, the consumption of glycerol by *S. clavuligerus* ORUN was increased by approximately 17.9%, as compared to that of the ORN strain (Fig. 4B). Accordingly, these results showed that the CA titer of *S. clavuligerus* ORUN generated through two-step mutagenesis using a reporter *neo* gene was improved with enhanced glycerol utilization.

### Gene Expression Analysis of glp Operon in ORUN Strain

**Fig. 5.**
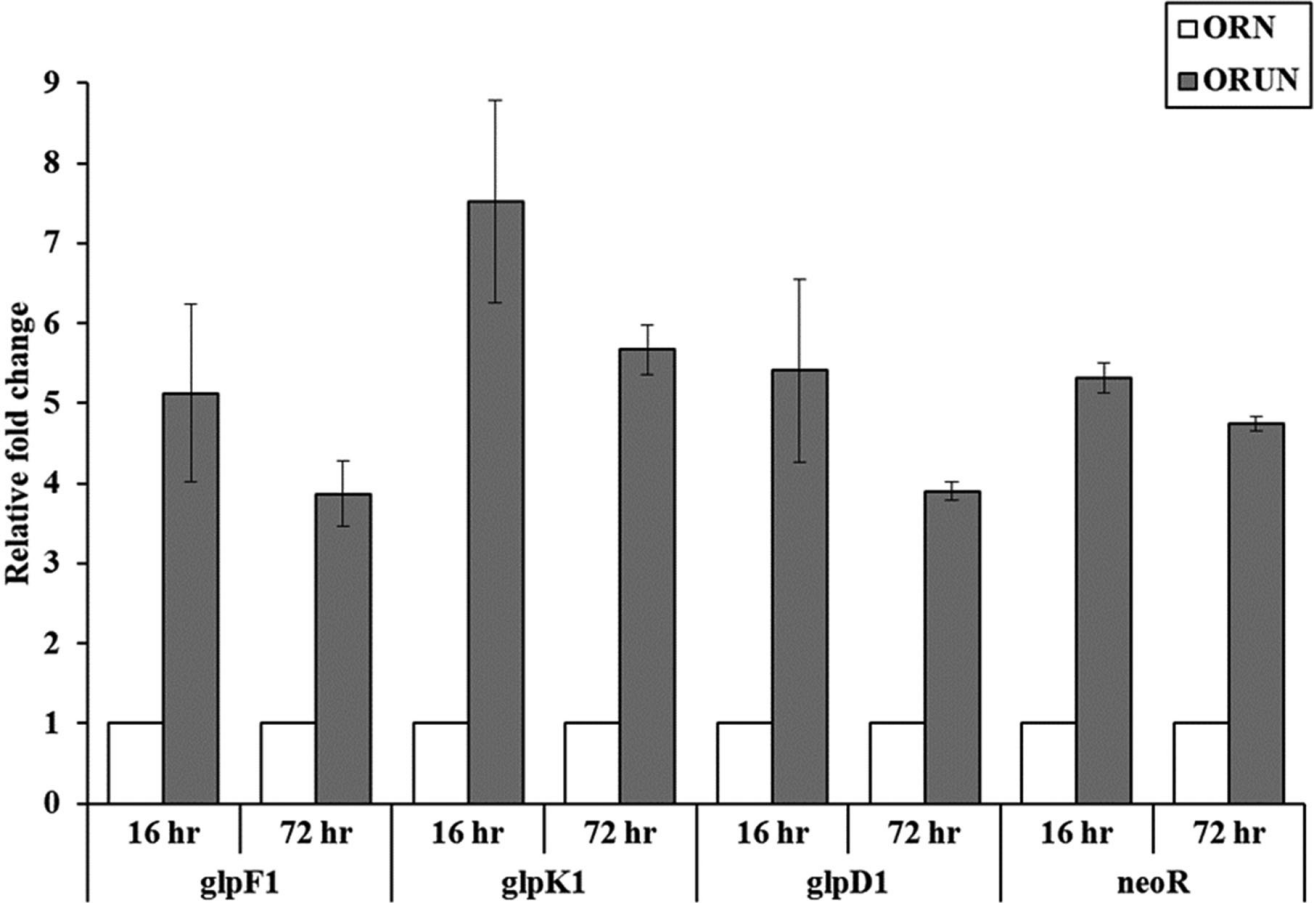
Relative expression level of *glp* operon in *S. clavuligerus* ORUN and OR at 16 and 72 h. The result is shown as fold-change of expression level in ORUN relative to the level of ORN, which was given the value of 1 for each gene.

Gene expression analysis was performed to investigate whether the increased CA production and glycerol utilization of the ORUN strain were caused by overexpression of the *glp* operon. The relative expression levels of the *glp* operon in the ORUN strain at 16 and 72 h of incubation time, respectively, were compared to those of the ORN strain (Fig. 5). Significant increases (approximately 5- to 7-fold) in the expressions of the *glp* operon in the ORUN strain at 16 h were observed, as compared to those of the ORN strain. The expression levels of the *glp* operon in the ORUN strain decreased at 72 h, as compared to those at 16 h, but they still showed remarkable increases (∼4- to 5-fold) as compared to those in the ORN strain. The degree of fold-change of *glpK1* expression of the ORUN strain was the highest over the entire incubation time: 7.5-fold and 5.7-fold at 16 and 72 h, respectively. The fold changes of both *glpF1* and *glpD1* expression in the ORUN strain were approximately 5.2- and 3.9-fold at 16 and 72 h, respectively, as compared to those of the ORN strain. Moreover, it was observed that the expression of *neo* fused downstream of *glpD1* in the ORUN strain increased at 16 and 72 h by approximately 5.3- and 4.7-fold, respectively, compared to that of the ORN strain. Because the *glp* operon and *neo* share an overlapping nucleotide at the TGA stop codon of *glpD1* and ATG start codon of *neo*, it can be expected that the promoterless *neo* is coordinately expressed under the control of a promoter upstream of *glpF1* (Fig. 2A) as reported previously (Wang et al., 2016).

In order to confirm that the overexpression of the *neo* gene in the ORUN strain actually increases the resistance against kanamycin, kanamycin resistance was evaluated in *S. clavuligerus* OR, ORN, and ORUN strains. The kanamycin strip test showed that the ORN strain produced a similar inhibition zone compared with the starting OR strain, but the ORUN strain showed a significantly smaller inhibition zone than those of the other mutant strains (Fig. S2). The MICs of kanamycin against OR, ORN, and ORUN strains by a kanamycin strip test were 0.38, 0.5, and 3 μg/ml, respectively. This indicated that the kanamycin resistance of ORUN strain was increased due to the increased expression of the *neo* gene. Taken together, these results demonstrate that a reporter strategy remains an effective approach for further improvement of high CA production in an industrial strain of *S. clavuligerus,* and the enhancement of CA titer and glycerol utilization of the ORUN strain resulted from increased co-transcription of the *glp* operon with a *neo* gene.

### Large-scale Production of CA by OR, ORN, and ORUN Strains

**Fig. 6.**
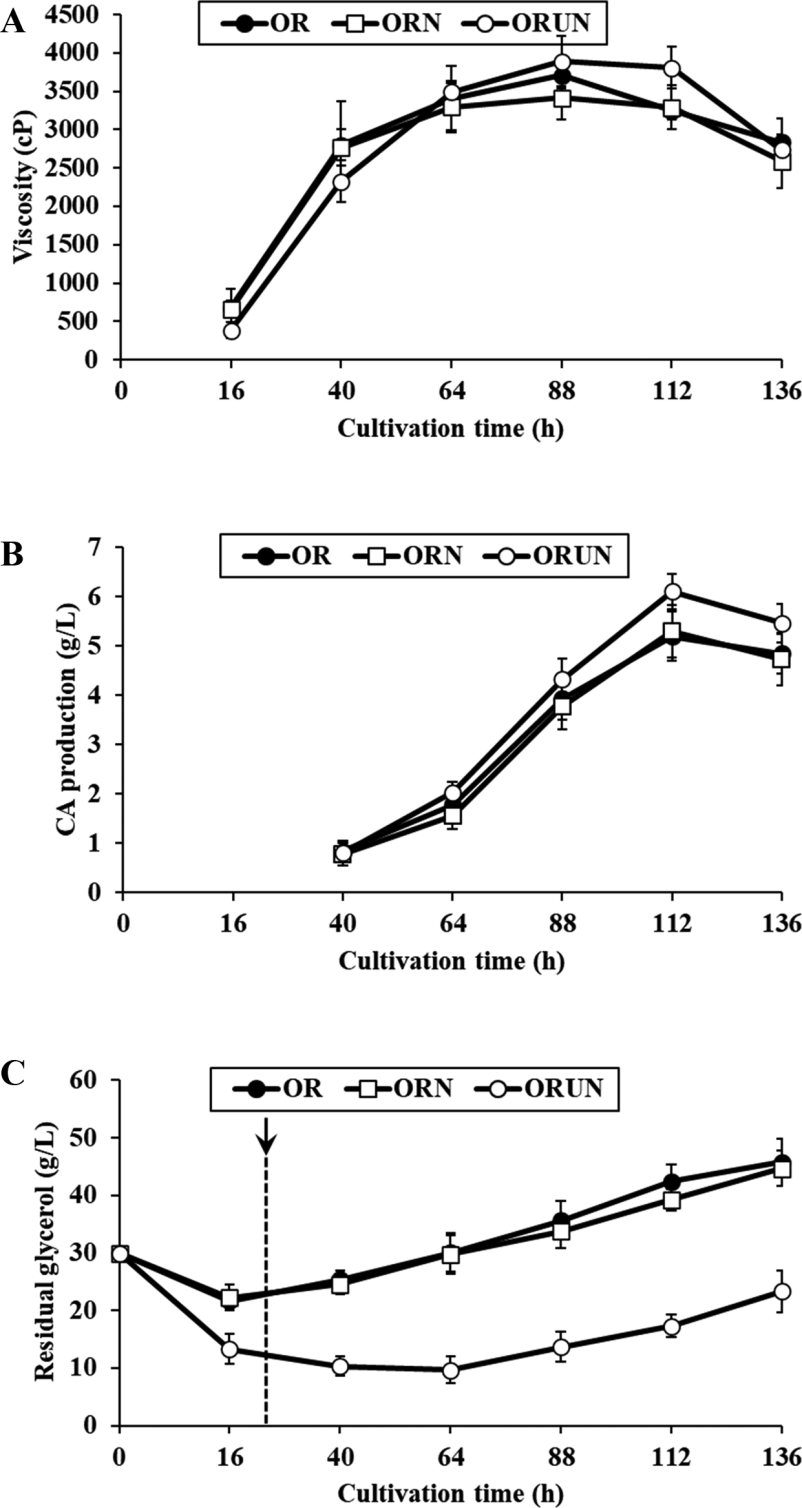
Time course of viscosity, clavulanic acid production, and residual glycerol from *S. clavuligerus* OR, ORN, and ORUN in 7 l fermenter. After 24 h incubation, 10 g/l/day of glycerol was fed continuously. The arrow indicates starting point (24 h) of continuous glycerol supplementation. (A) Viscosity; (B) clavulanic acid production; (C) residual glycerol.

The production of CA, the residual amount of glycerol, and the growth of the OR, ORN, and ORUN strains were monitored in a 7 l fermenter over a 136 h incubation period (Fig. 6). The growth of the OR and ORN strains substantially increased between 16 and 40 h and reached stationary phase after 64−88 h (Fig. 6A). CA production was increased in the late exponential and stationary growth phases, and the maximum CA titer was observed at 112 h in both of the OR and ORN mutant strains (Fig. 6B). The residual glycerol in the culture of the OR and ORN strains was notably decreased at early exponential phase and increased over the initial level (30 g/l) at 64 h with additional glycerol supplementation; 10 g/l/day of glycerol was fed continuously after 24 h of incubation (Fig. 6C).

In the case of ORUN, cell growth was slightly delayed between 16 and 40 h, as compared to the other mutants but reached growth levels greater than those of the other mutants after 64 h (Fig. 6A). The residual glycerol in the culture of the ORUN strain was remarkably lower after 136 h of incubation time, as compared to those of other mutant strains (Fig. 6C), which suggests that the ORUN strain utilized more glycerol than other mutant strains. The glycerol consumption of the ORUN strain was consistently increased between 0 and 64 h and moderately decreased after 64 h. It was observed that CA production was moderately increased between 40 and 64 h and increased rapidly after 64 h in the ORUN strain. The maximum CA titer in ORUN was increased by approximately 17.4% (6.11 ± 0.36 g/l) at 112 h, as compared to that of the OR strain (Fig. 6B). Based on these results, we confirmed the crucial effect of glycerol utilization of *S. clavuligerus* on CA production and cell growth in large-scale fermentation.

While the CA production by the ORUN strain in a flask culture was increased by approximately 39.1% (Fig. 4A), the maximum CA production of this mutant in the fermenter was enhanced by approximately 15.1% over that of the ORN strain (Fig. 6B). The conditions for fermenter culture need to be optimized for further improvement of CA titer in the mutant strains. Nevertheless, the resulting ORUN strain displayed a 9.8-fold (5.21 ± 0.26 g/l) increase in CA production in flask culture, as compared to that of wild-type reported in our previous study (Cho et al., 2019). The CA titer (6.11 ± 0.36 g/l) by the ORUN strain in the fermenter culture is one of the highest CA titers produced by *S. clavuligerus* that has been recently reported (Cho et al., 2019; Kizildoğan et al., 2017).

These results demonstrate that further enhancement of CA titer in randomly mutagenized industrial strains of *S. clavuligerus* can be achieved by overexpression of the *glp* operon and confirmed an important role of glycerol on the high production of CA and cell growth of *S. clavuligerus*. Our results also showed that reporter-guided mutant selection can be an efficient method for further enhanced production of secondary metabolites through random mutagenesis of an industrial strain.

## Supplementary Material

kuab004_Supplemental_File

## References

[bib1] Baker C. N. , Stocker S. A., Culver D. H., Thornsberry C. (1991). Comparison of the E Test to agar dilution, broth microdilution, and agar diffusion susceptibility testing techniques by using a special challenge set of bacteria. Journal of Clinical Microbiology, 29(3), 533–538. 2037671 10.1128/jcm.29.3.533-538.1991 PMC269813

[bib2] Baños S. , Pérez-Redondo R., Koekman B., Liras P. (2009). Glycerol utilization gene cluster in *Streptomyces clavuligerus*. Applied and Environmental Microbiology, 75(9), 2991–2995. 19286797 10.1128/AEM.00181-09 PMC2681673

[bib3] Bierman M. , Logan R., O'Brien K., Seno E. T., Rao R. N., Schoner B. E. (1992). Plasmid cloning vectors for the conjugal transfer of DNA from *Escherichia coli* to *Streptomyces* spp. Gene, 116, 43–49. 1628843 10.1016/0378-1119(92)90627-2

[bib4] Bird A. E. , Bellis J. M., Gasson B. C. (1982). Spectrophotometric assay of clavulanic acid by reaction with imidazole. Analyst, 107(1279), 1241–1245.

[bib5] Chen K. C. , Lin Y. H., Wu J. Y., Hwang S. C. J. (2003). Enhancement of clavulanic acid production in *Streptomyces clavuligerus* with ornithine feeding. Enzyme and Microbial Technology, 32(1), 152–156.

[bib6] Cho H. S. , Jo J. C., Shin C. H., Lee N., Choi J. S., Cho B. K., Roe J. H., Kim C. W., Kwon H. J., Yoon Y. J. (2019) Improved production of clavulanic acid by reverse engineering and overexpression of the regulatory genes in an industrial *Streptomyces clavuligerus* strain. Journal of Industrial Microbiology & Biotechnology, 46, 1205–1215. 31165280 10.1007/s10295-019-02196-0

[bib7] Daskalaki V. M. , Kondarides D. I. (2009). Efficient production of hydrogen by photo-induced reforming of glycerol at ambient conditions. Catalysis, 144, 75–80.

[bib8] Elander R. P. (2003). Industrial production of β-lactam antibiotics. Applied Microbiology and Biotechnology, 61, 385–392. 12679848 10.1007/s00253-003-1274-y

[bib9] Guo D. , Zhao Y., Yang K.. (2013). Coordination of glycerol utilization and clavulanic acid biosynthesis to improve clavulanic acid production in *Streptomyces clavuligerus*. Science China Life Science, 56(7), 591–600. 10.1007/s11427-013-4507-z 23832248

[bib10] Hamed R. B. , Gomez-Castellanos J. R., Henry L., Ducho C., McDonough M. A., Schofield C. J. (2013). The enzymes of β-lactam biosynthesis. Natural Product Reports, 30(1), 21–107. 23135477 10.1039/c2np20065a

[bib11] Jensen S. E. (2012). Biosynthesis of clavam metabolites. Journal of Industrial Microbiology & Biotechnology, 39(10), 1407–1419. 22948564 10.1007/s10295-012-1191-0

[bib12] Jung W. S. , Yoo Y. J., Park J. W., Park S. R., Han A. R., Ban Y. H., Kim E. J., Kim E., Yoon Y. J. (2011). A combined approach of classical mutagenesis and rational metabolic engineering improves rapamycin biosynthesis and provides insights into methylmalonyl-CoA precursor supply pathway in *Streptomyces hygroscopicus* ATCC 29253. Applied Microbiology and Biotechnology, 91(5), 1389–1397. 21655985 10.1007/s00253-011-3348-6

[bib13] Kieser T. , Bibb M. J., Buttner M. J., Chater K. F., Hoopwood D. A. (2000). Practical Streptomyces genetics. John Innes Centre.

[bib14] Kim S. J. , Kim J. O., Shin C. H., Park H. W., Kim C. W. (2009). An approach to strain improvement and enhanced production of clavulanic acid in *Streptomyces clavuligerus*. Bioscience, Biotechnology, and Biochemistry, 73(1), 160–164. 19129630 10.1271/bbb.80569

[bib15] Kizildoğan A. K. , Jaccard G. V., Mutlu A., Sertdemir I., Özcengiz G. (2017). Genetic engineering of an industrial strain of *Streptomyces clavuligerus* for further enhancement of clavulanic acid production. Turkish Journal of Biology, 1(2), 342–353.

[bib16] Li S. , Wang W., Li X., Fan K., Yang K. (2015). Genome-wide identification and characterization of reference genes with different transcript abundances for *Streptomyces coelicolor*. Scientific Reports, 5, 15840. 26527303 10.1038/srep15840 PMC4630627

[bib17] Medema M. H. , Alam M. T., Heijne W. H., van den Berg M. A., Müller U., Trefzer A., Bovenberg R. A. L., Breitling R., Takano E. (2011). Genome-wide gene expression changes in an industrial clavulanic acid overproduction strain of *Streptomyces clavuligerus*. Microbial Biotechnology, 4(2), 300–305. 21342474 10.1111/j.1751-7915.2010.00226.x PMC3818869

[bib18] Mo S. , Kim D. H., Lee J. H., Park J. W., Basnet D. B., Ban Y. H., Yoo Y. J., Chen S. W., Park S. R., Choi E. A., Kim E., Jin Y. Y., Lee S. K., Park J. Y., Liu Y., Lee M. O., Lee K. S., Kim S. J., Kim D., Yoon Y. J. (2011). Biosynthesis of the allylmalonyl-CoA extender unit for the FK506 polyketide synthase proceeds through a dedicated polyketide synthase and facilitates the mutasynthesis of analogues. Journal of the American Chemical Society, 133, 976–985. 21175203 10.1021/ja108399b PMC3030623

[bib19] Neves A. A. , Pereira D. A., Vieira L. M., Menzes J. C. (2000). Real time monitoring biomass concentration in *Streptomyces clavuligerus* cultivations with industrial media using a capacitance probe. Journal of Biotechnology, 84, 45–52. 10.1016/s0168-1656(00)00325-4 11035186

[bib20] Paradkar A. (2013). Clavulanic acid production by *Streptomyces clavuligerus*: Biogenesis, regulation and strain improvement. Journal of Antibiotics, 66(7), 411. 23612724 10.1038/ja.2013.26

[bib21] Qin R. , Zhong C., Zong G., Fu J., Pang X., Cao G. (2017). Improvement of clavulanic acid production in *Streptomyces clavuligerus* F613-1 by using a *claR*-*neo* reporter strategy. Electronic Journal of Biotechnology, 28, 41–46.

[bib22] Rowlands R. T. (1984). Industrial strain improvement: Mutagenesis and random screening procedures. Enzyme and Microbial Technology, 6(1), 3–10.

[bib23] Song L. , Laguerre S., Dumon C., Bozonnet S., O'Donohue M. J. (2010). A high-throughput screening system for the evaluation of biomass-hydrolyzing glycoside hydrolases. Bioresource Technology, 101(21), 8237–8243. 20579873 10.1016/j.biortech.2010.05.097

[bib24] Tahlan K. , Jensen S. E. (2013). Origins of the β-lactam rings in natural products. Journal of Antibiotics, 66(7), 401. 23531986 10.1038/ja.2013.24

[bib25] Tian T. , Salis H. M. (2015). A predictive biophysical model of translational coupling to coordinate and control protein expression in bacterial operons. Nucleic Acids Research, 43(14), 7137–7151. 26117546 10.1093/nar/gkv635 PMC4538824

[bib26] Ünsaldı E. , Kurt-Kızıldoğan A., Voigt B., Becher D., Özcengiz G. (2017). Proteome-wide alterations in an industrial clavulanic acid producing strain of *Streptomyces clavuligerus*. Synthetic and Systems Biotechnology, 2(1), 39–48. 29062960 10.1016/j.synbio.2016.10.003 PMC5625738

[bib27] Wang Y. , Tao Z., Zheng H., Zhang F., Long Q., Deng Z., Tao M. (2016). Iteratively improving natamycin production in *Streptomyces gilvosporeus* by a large operon-reporter based strategy. Metabolic Engineering, 38, 418–426. 27746324 10.1016/j.ymben.2016.10.005

[bib28] Xiang S. H. , Li J., Yin H., Zheng J. T., Yang X., Wang H. B., Luo J. L., Bai H., Yang K. Q. (2009). Application of a double-reporter-guided mutant selection method to improve clavulanic acid production in *Streptomyces clavuligerus*. Metabolic Engineering, 11(4–5), 310–318. 19584003 10.1016/j.ymben.2009.06.003

