# Supplementary material for: Enhanced production of clavulanic acid by improving glycerol utilization using reporter-guided mutagenesis of an industrial Streptomyces clavuligerus strain
Source: J Ind Microbiol Biotechnol. 2021 Jan 25;48(3-4):kuab004. doi: 10.1093/jimb/kuab004 (PMC9113135; doi:10.1093/jimb/kuab004)
Supplement: kuab004_Supplemental_File [file kuab004_supplemental_file.docx]

*Supplementary Material*

**Enhanced Production of Clavulanic Acid by Improving Glycerol Utilization Using Reporter-Guided Mutagenesis of an Industrial *Streptomyces clavuligerus* Strain**

Chang-Hun Shin^1^, Hang Su Cho^2^, Hyung-Jin Won^3^, Ho Jeong Kwon^2*^, Chan-Wha Kim^1*^, and Yeo Joon Yoon^4*^

*^1^Department of Biotechnology, College of Life Sciences and Biotechnology, Korea University, Seoul 02841, Republic of Korea*

^2^Department of Biotechnology, College of Life Science and Biotechnology, Yonsei University, Seoul 03722, Republic of Korea

*^3^Fermentation Technology Team, Research Institute of CKD Bio, Ansan 15604, Republic of Korea*

*^4^Natural Products Research Institute, College of Pharmacy, Seoul National University, Seoul 08826, Republic of Korea*

* Corresponding author: Yeo Joon Yoon, Chan-Wha Kim, and Ho Jeong Kwon

+82-2-880-2379

[yeojoonyoon@snu.ac.kr](mailto:yeojoonyoon@snu.ac.kr), [cwkim@korea.ac.kr](mailto:cwkim@korea.ac.kr), and kwonhj@yonsei.ac.kr

Chang-Hun Shin and Hang Soo Cho have contributed equally to this work.

**Table S1** Primer sequences used for RT-PCR analysis used in this study.

| **Primers** | **Sequences (5’-3’)** | **Description** |
| --- | --- | --- |
| glpF1-F | TCATGTCCCACCTCAAGGAC | Forward primer for *glpF1* |
| glpF1-R | GATCTCGGTGACGAGGTTCT | Reverse primer for *glpF1* |
| glpK1-F | TGGGTCATCTGGAACCTGAC | Forward primer for *glpK1* |
| glpK1-R | GGAGCTGAGGATCTTCTCGT | Reverse primer for *glpK1* |
| glpD1-F | CGATCGACAAGTACCGCATC | Forward primer for *glpD1* |
| glpD1-R | CTTCTCCGTCACCGTCACAT | Reverse primer for *glpD1* |
| neo-F | GGCTGCTATTGGGCGAAGTG | Forward primer for *neo* |
| neo-R | GATGTTTCGCTTGGTGGTCG | Reverse primer for *neo* |
| hrdB-F | GCGGTGGAGAAGTTCGACTA | Forward primer for *hrdB* |
| hrdB-R | CACATGGTCGAGGTCATCAA | Reverse primer for *hrdB* |

AAGCTTGTGACCGAGGGCTCCGGCGGCATGCTGTCGGTGGCGGGCGGCAAGTGGACGACCTTCCGCCACATCGGCCGCACCATCATGAACAAGCTCGCCGAGCTGCCCGGCCGGCCGCTCGCCGAGGACATGGAGCCGATGTCGCGGCTGCCGAAGAAGGTGCCGCTGCCCGGTATAGCCAACCCGGACGCGGTGGCGCACCGGTTGCTCGTCGACGGCGGTACGCCCGGTCCCCGGATGGCGGCGGACACCGCCCGCCACCTGGCGACCCACTACGGCTCGCTGGCGTTCGACATCGCCCGGCTCGCCAACGACGACCCGGCGCTCGCGGAGCGCGTCCACCCGGACGCCCCCGAGATCTGGGCGCAGGTCGTCTACGCGCGGGACCACGAGTGGGCCGAGACGGCGGACGACGTGCTGCGCCGCCGGACCACGCTGACGATCCGCGGTCTCGCGACGGACGAGATCCGCGGCAGGGTGGACGGTCTGCTCAAGGACCGCGCCTG**ATGATTGAACAAGATGGATTGCACGCAGGTTCTCCGGCCGCTTGGGTGGAGAGGCTATTCGGCTATGACTGGGCACAACAGACAATCGGCTGCTCTGATGCCGCCGTGTTCCGGCTGTCAGCGCAGGGGCGCCCGGTTCTTTTTGTCAAGACCGACCTGTCCGGTGCCCTGAATGAACTGCAGGACGAGGCAGCGCGGCTATCGTGGCTGGCCACGACGGGCGTTCCTTGCGCAGCTGTGCTCGACGTTGTCACTGAAGCGGGAAGGGACTGGCTGCTATTGGGCGAAGTGCCGGGGCAGGATCTCCTGTCATCTCACCTTGCTCCTGCCGAGAAAGTATCCATCATGGCTGATGCAATGCGGCGGCTGCATACGCTTGATCCGGCTACCTGCCCATTCGACCACCAAGCGAAACATCGCATCGAGCGAGCACGTACTCGGATGGAAGCCGGTCTTGTCGATCAGGATGATCTGGACGAAGAGCATCAGGGGCTCGCGCCAGCCGAACTGTTCGCCAGGCTCAAGGCGCGCATGCCCGACGGCGAGGATCTCGTCGTGACCCATGGCGATGCCTGCTTGCCGAATATCATGGTGGAAAATGGCCGCTTTTCTGGATTCATCGACTGTGGCCGGCTGGGTGTGGCGGACCGCTATCAGGACATAGCGTTGGCTACCCGTGATATTGCTGAAGAGCTTGGCGGCGAATGGGCTGACCGCTTCCTCGTGCTTTACGGTATCGCCGCTCCCGATTCGCAGCGCATCGCCTTCTATCGCCTTCTTGACGAGTTCTTCTGA**ACACGGACGCGCCACGGCCGGAGGCCGGGCCCGGGACACCGCTCCCGGGCCCGGCCTCCGGTCCGTCCGGGCCCCGTTGCCCCGGTCTCCCCCTCGTCGGCGGACGGGTCCACGCGCTGGTCCCGTCCGTGTCGGGCGCCGTCCCACGCTGTACGCTCAGACCTCCGATGACTCATGGTTCATGAGGACGAGGGGGCGCGGCATGGCGGTCACCGACGAGGCGATCGAGAAGATCAAGGGAATGATCGTCTCGGGGGCGCTGCGCCCCGGGGACCGGCTGCCCAAGGAGAGCGAGCTGGCCGCCGGTCTCGGGCTGTCCCGCAATTCGCTGCGGGAGGCGGTGCGGGCGCTGTCACTGATCCGGATTCTGGATGTGCGGCAGGGCGACGGCACCTATGTGACCAGTCTCGACCCGCAGTTGCTGCTGGAGGCGCTGAGCTTCGTGGTGGACTTCCACCGCGACGACACGGTGCTGGA

GTTCCTGGCCGTACGGCGCATCCTCTAGA

**Fig. S1** Nucleotide sequences for *neo* gene including flanking regions. *neo* gene is shown by bold and enzyme sites are underlined.


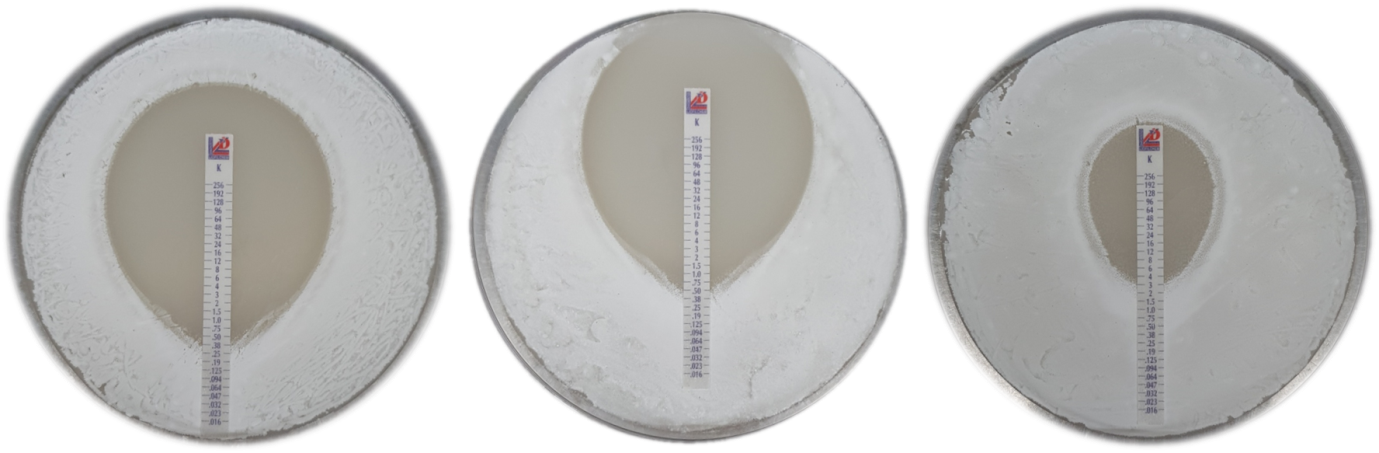


**Fig. S2** Minimum inhibitory concentration test for *S. clavuligerus* OR (left), ORN (center), and ORUN (right) against kanamycin.
